# Joint Skewness and Its Application in Unsupervised Band Selection for Small Target Detection

**DOI:** 10.1038/srep09915

**Published:** 2015-04-15

**Authors:** Xiurui Geng, Kang Sun, Luyan Ji, Hairong Tang, Yongchao Zhao

**Affiliations:** 1 Key Laboratory of Technology in Geo-spatial Information Processing and Application System, Institute of Electronics, Chinese Academy of Sciences, Beijing, China; 2 Ministry of Education Key Laboratory for Earth System Modelling, Centre for Earth System Science, Tsinghua University, Beijing, China

## Abstract

Few band selection methods are specially designed for small target detection. It is well known that the information of small targets is most likely contained in non-Gaussian bands, where small targets are more easily separated from the background. On the other hand, correlation of band set also plays an important role in the small target detection. When the selected bands are highly correlated, it will be unbeneficial for the subsequent detection. However, the existing non-Gaussianity-based band selection methods have not taken the correlation of bands into account, which generally result in high correlation of obtained bands. In this paper, combining the third-order (third-order tensor) and second-order (correlation) statistics of bands, we define a new concept, named joint skewness, for multivariate data. Moreover, we also propose an easy-to-implement approach to estimate this index based on high-order singular value decomposition (HOSVD). Based on the definition of joint skewness, we present an unsupervised band selection for small target detection for hyperspectral data, named joint skewness band selection (JSBS). The evaluation results demonstrate that the bands selected by JSBS are very effective in terms of small target detection.

Benefiting from the significant development in last several decades, hyperspectral imaging sensors can now simultaneously obtain hundreds of bands of the ground objects. As a result, hyperspectral data sets provide much more bands and therefore a much better discrimination among similar ground cover classes than traditional multispectral imageries^1^,^2^. However, the bands are usually high-correlated due to the high spectrum resolution which results in great redundancy in hyperspectral data sets. Moreover, computational requirements to deal with large hyperspectral data sets might be prohibitive. Therefore, dimensionality reduction has been received considering attention which can resolve this problem well.

In general, two broad categories of dimensional reduction approaches are frequently used for hyperspectral data, namely, feature extraction and feature selection (or band selection in hyperspectral field). Feature extraction techniques generate a small set of features based on functional mappings of the original ones. Methods of type include principal components analysis (PCA)^3^ maximum noise fraction (MNF)^4^, independent components analysis (ICA)^5^ and some recently reported methods such as^6^,^7^,^8^. But the features obtained by these methods are generally lack of physical interpretation due to the transformation to the original features. Band selection (BS), on the other hand, aims to select subset of features from the original larger/full band set. The features obtained by BS techniques are of specific physical meaning since no transformation is involved.

Band selection can be performed in either supervised or unsupervised manners^1^. Supervised BS methods require some priori knowledge such as training samples or target signatures, for instance,^9^,^10^,^11^,^12^,^13^,^14^. However, these training samples are sometimes not available in practice since the acquisition of reliable samples is very expensive in terms of both time and money^15^,^16^. In this case, the unsupervised methods which can generally provide acceptable performance regardless the types of ground cover classes, are necessary. Many unsupervised BS techniques have been reported in literatures, most of which are based on information evaluation means. These methods first defines some criterion functions (cost functions), for instance, variances^17^,^18^, information entropy^19^, information divergence^20^ and correlation^15^,^21^, followed by searching for the optimal (suboptimal) band subset. Some other unsupervised BS methods can be seen in references^22^,^23^,^24^,^25^,^26^.

As for these BS methods, few of them are specially proposed for target detection despite target detection is one of the most important applications of hyperspectral remote sensing. We therefore focus our attention on unsupervised BS methods for target detection in this paper. As is well known, the artificial small targets generally drive data clouds deviating from Gaussian distribution. Consequently, the bands with more non-Gaussianity possibly contain more information on artificial small targets. Some methods have employed non-Gaussianity as BS criterion function, for instance, information divergence band selection (IDBS)^20^ prioritizing the bands according to how far they deviates from the corresponding Gaussian distribution. The higher the ID value, the higher weight of a band. Du et al.^27^ have proposed an unsupervised BS methods based on FastICA. This method, instead of transforming the original hyperspectral images, evaluates the weight matrix to observe how each band contributes to the ICA unmixing procedure. It compares the average absolute weight coefficients of individual spectral bands and selects bands that contain more information. However, both the two methods have not taken the correlation of bands into account which may result in high correlation of the selected bands.

In this paper, we integrate the non-Gaussianity and correlation of the bands by defining *joint skewness* (JS) for multivariate data, which not only characterizes the non-Gaussianity of a band subset but also depresses the correlation. More specifically, we known that, the determinant of the covariance matrix of hyperspectral data can be regarded as *joint second-order statistics*^17^ (briefly reviewed in section 2.1). We extend this idea to third-order statistics of the bands and employ the hyperdeterminant of the coskewness tensor to measure the joint skewness. However, the calculation of the tensor hyperdeterminant is quite difficult especially for the tensor with size larger than 2 × 2 × 2^28^. Interestingly, by introducing the idea of high-order of singular value decomposition (HOSVD), we can conveniently obtain the approximate value of JS for band subset via transforming the hyperdeterminant of tensor to the product of the singular value derived from HOSVD. Furthermore, based on JS defined, we present an unsupervised BS method for artificial small targets detection, named joint skewness based band selection (JSBS). In order to save subset search time, the subset selection method, sequential background selection (SBS) is employed.

## Results

To evaluate the performance of JSBS, we conduct some tests based on both simulated data and real hyperspectral data in this section. Three unsupervised band selection methods, namely, maximum ellipsoid volume (MEV)^17^, information divergence band selection (IDBS)^20^, ICA based band selection (ICABS)^27^ and supervised band selection method, variable-number variable-band selection (VNVBS)^29^ are also compared with JSBS.

### Evaluation with simulated data

#### Test1: the relationship between HOSVD and the hyperdeterminant

**Figure 1:**
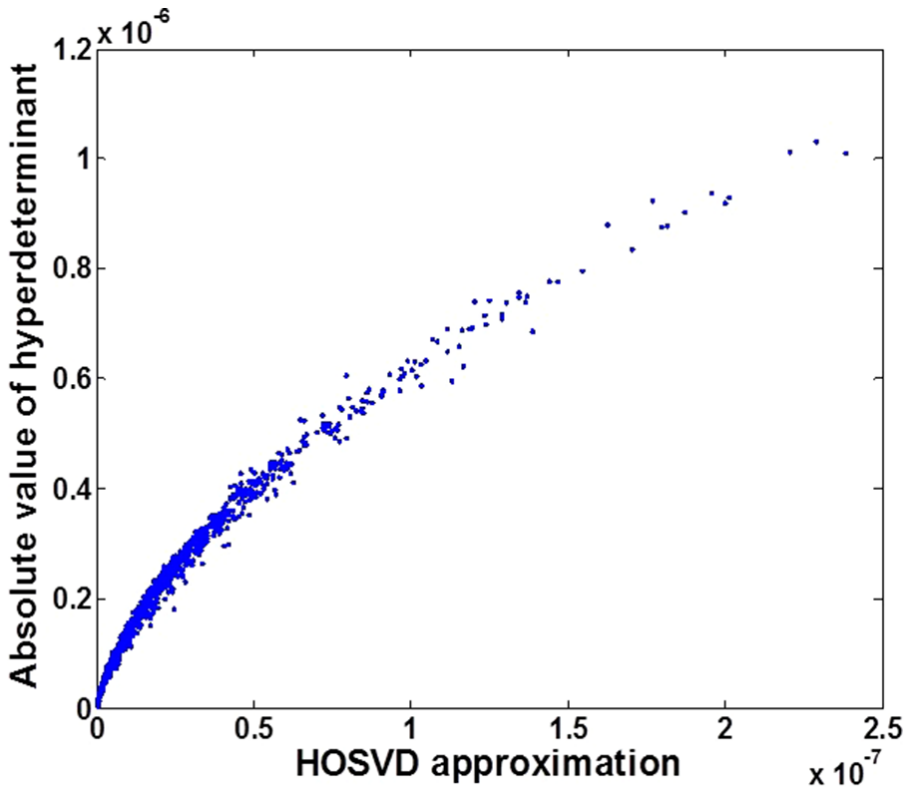
HOSVD approximation for hyperdeterminant of tensor.

In this section, we investigate the relationship between the hyperdeterminant of third-order cumulant and its approximation 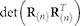 derived from HOSVD using simulated data. Since only the hyperdeterminant of tensor with size of 2 × 2 × 2 can be computed based on equation (2), we randomly generate 1000 images. Each image contains 2 bands with 1000 pixels in each band. The pixels satisfy uniform distributions between [0, 1], generated by function rand in Matlab software. Then the corresponding third-order cumulant and its hyperdeterminant 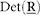 are computed. Meanwhile, the approximation values 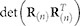 are also calculated. The results are plotted in Figure 1.

From Figure 1 we can see that, 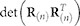 has an obviously positive correlation with 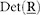 although they do not close to each other in absolute value. The correlation coefficient between the two groups of values is 0.9675 which indicates that our proposed approximation is very effective.

#### Test2: The superiority of the JSBS

In this section, we designed a simulated experiment to compare the performances of some unsupervised band selection methods, namely, MEV, IDBS, ICABS and JSBS.

**Figure 2:**
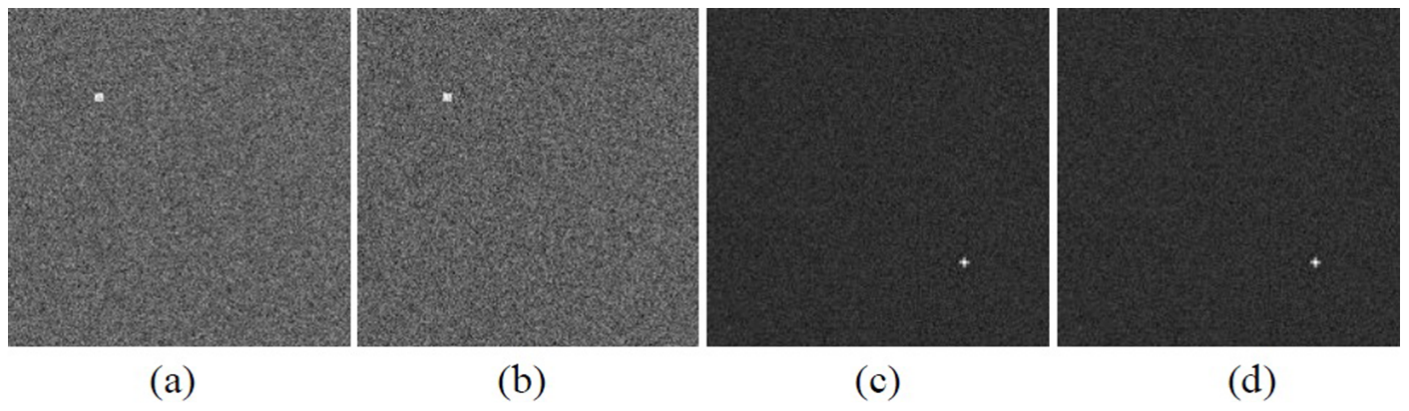
Simulated data: (a) band 1, (b) band 2, (c) band 3 and (d) band 4.

The use of joint skewness enables JSBS to take all the different small targets into account simultaneously. In order to verify the superiority of JSBS, we generate the following simulated data. The simulated data consists of 4 bands with size of 200*200 pixels. Two artificial small targets (see Figure 2) are added to the data set. One of the targets shows large difference with the background in the first two bands (Figure 2 a, b) while the other target is different from the background mainly in the last two bands (Figure 2 c, d).

All the backgrounds follow Gaussian distributions, but we also make some purposeful modifications. Specifically, band 1 and band 2 are of large variances, low skewness and low correlations, while band 3 and band 4 are of low variances, large skewness and high correlations. The specifications of the data are listed in Table 1.

In order to compare the performance of MEV, IDBS, ICABS and JSBS, we selected two bands from the simulated data using these methods. The band selection results are shown in Table 2.

From the results, we can see that only the subset selected by JSBS contains the information of both targets. More specifically, MEV prefers to the subset with large variance and low correlation, therefore, it selected band 1 and band 2 which only contain the information of the first target. This result indicates that the subset with the maximum 2nd-order statistics may be not suitable for small target detection. On the other hand, although IDBS and ICABS try to select non-Gaussian subset, they do not take the correlation of the bands into account. Therefore, although band 3 and band 4 are of high correlation, they are still selected by IDBS and ICABS. As a result, both IDBS and ICABS ignored the first target. As for JSBS, it takes both non-Gaussianity and correlation into account and tries to select the subset with maximum joint skewness. Consequently, it selected band 2 and band 4 which contains both targets of interest. This test verifies that JSBS can take the multiple small targets into account while MEV, IDBS and ICABS may ignore partial targets.

### Cuprite data

**Figure 3:**
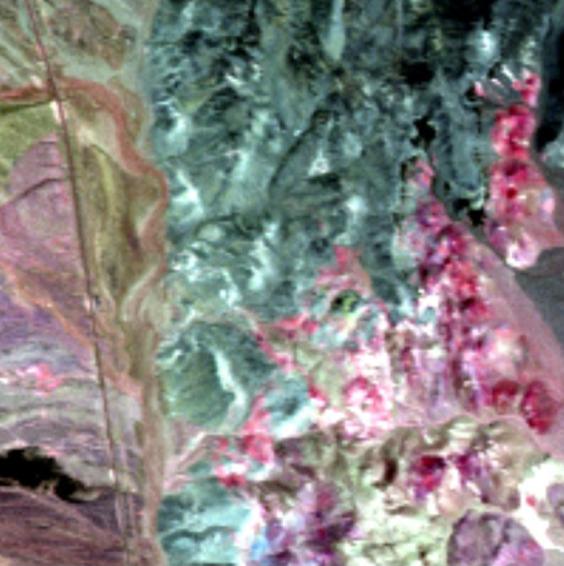
The subscene used in this test, containing Nontronite and Buddingtonite.

In this section, the algorithms were applied to real hyperspectral image acquired by Airborne Visible Infrared Imaging Spectrometer (AVIRIS) on June 19 in 1997 over Cuprite mining site, Nevada. The data is widely used in remote sensing experiments because of the detailed corresponding ground truth and the free access. The data used in this experiment is attached in ENVI software, consisting of a total of 50 bands ranging from 1991 nm to 2479 nm. We cropped a subscene (shown in Figure 3) which contains two types of mineral with low probability distribution, namely Nontronite and Buddingtonite for this test. We use this test to investigate if these methods can simultaneously take different targets into account.

**Figure 4:**
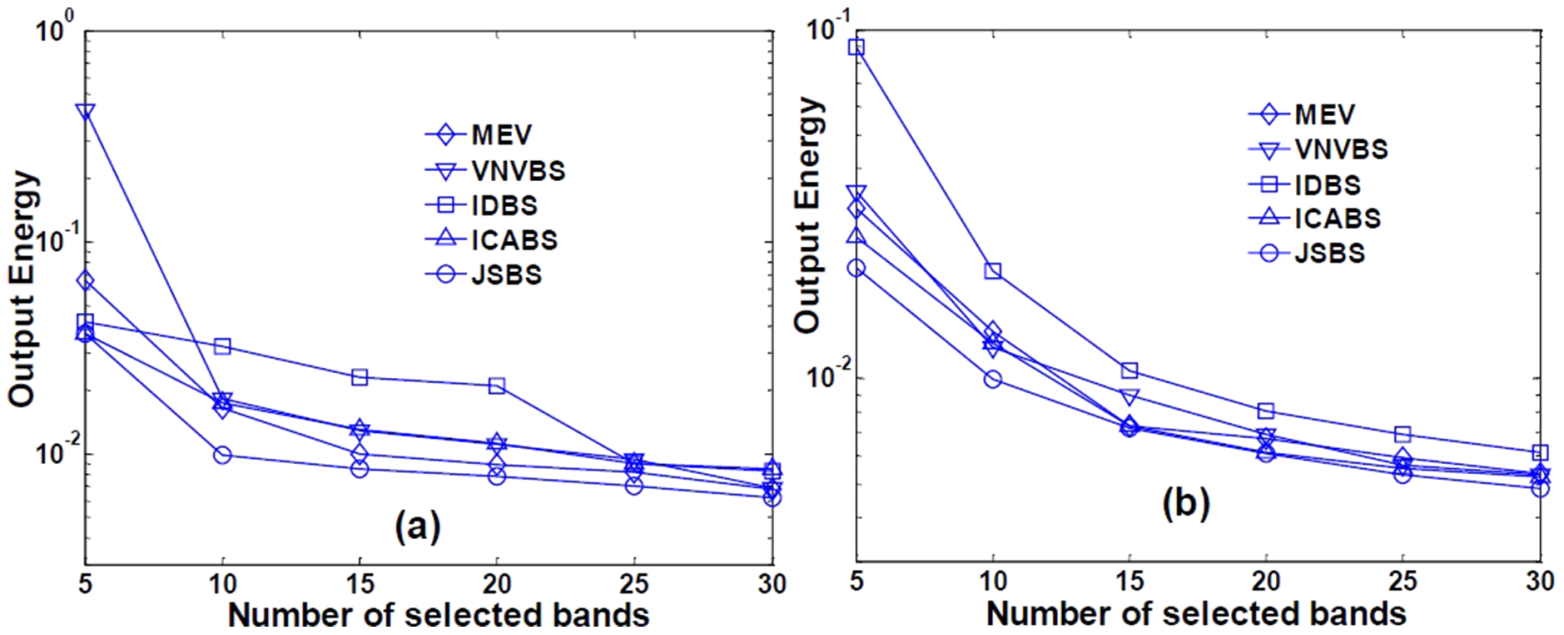
CEM output energy using different subsets (a) Nontronite and (b) Buddingtonite.

We selected 5, 10, 15, 20, 25 and 30 bands from the image by these methods, then the selected subset are used for CEM target detection. The representative signatures of the two targets can be selected directly from the image by endmember extraction methods, such as fast gram determinant algorithm (FGDA)^30^ and gaussian elimination method (GEM)^31^. In this paper, we use the efficient technique, GEM, to select the spectrums. In addition, the mean vector of the image is employed as the reference signature required in VNVBS. To quantitatively evaluate the performance of these methods, we adopt the objective function of CEM, output energy as the comparison index, which is expected to be as low as possible. The output energy derived from these methods is shown in Figure 4.

As can be seen from Figure 4, the output energies of all the methods are monotonously decreasing with the increasing number of selected bands, which is consistent with the conclusion in^32^. The subset selected by the proposed JSBS is the most discriminative in terms of both targets, verifying the conclusion in Test 2. Although IDBS and ICABS also prefer the bands with high non-gaussianity, they do not suppress the correlation of bands. Therefore, the obtained subsets have poor performance in target detection. MEV considered the mainly the background of the image and paid little attention to the small targets. VNVBS is a supervised method, which concentrates on the separation between target and reference signatures and neglects the structure of the background.

**Figure 5:**
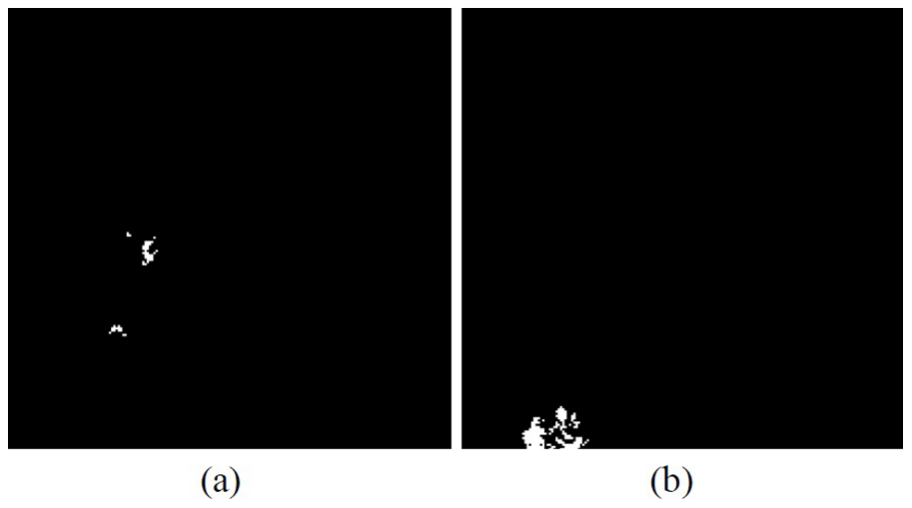
Manually-made ground truth for (a) Nontronite and (b) Buddingtonite.

**Figure 6:**
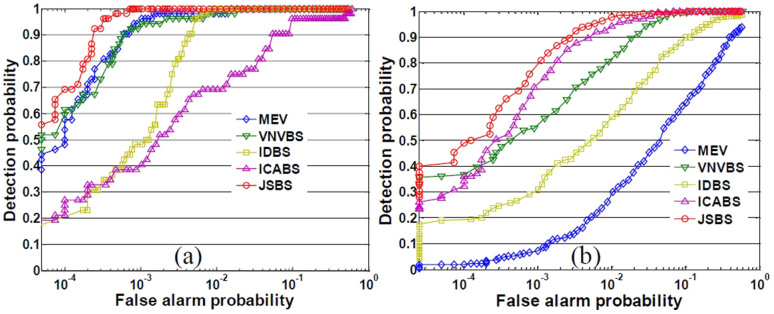
ROC curves for (a) Nontronite and (b) Buddingtonite.

In order to further compare the performances of these methods, we use receiver operating characteristic (ROC) as the measurement. We manually made the ground truth maps (shown in Figure 5) for the two minerals according to the fully constrained unmixing^33^ results and the ground investigation^34^ since there is no pixel-level ground truth. Then we used these methods to select 10 bands which were involved in the subsequent CEM detection. The ROC curves for the CEM detection results using different subsets are shown in Figure 6.

The ROC curves in Figure 6 demonstrate that, the subset selected by JSBS has the best detection performance in terms both Nontronite and Buddingtonite since it has the highest detection probability at the same false alarm ratio. As an unsupervised band selection method, JSBS can pay attention to different targets simultaneously. The other methods did not have robust performances since they have not taken the correlation into account (IDBS and ICABS), mainly consider the background information (MEV) or neglected the background information (VNVBS).

### Wuxi data

**Figure 7:**
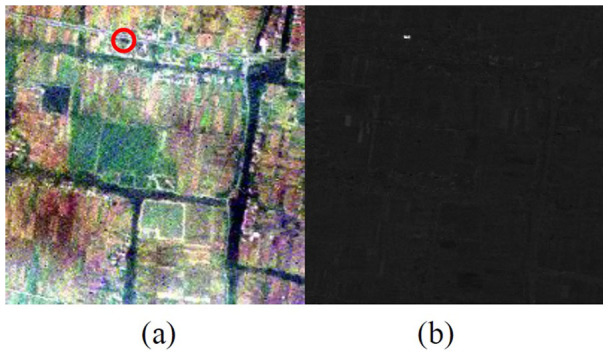
(a) True color of the image (b) CEM results of the target from full bands.

This data was obtained by Operational Modular Imaging Spectrometer I (OMIS-I) in Wuxi, China. It contains a total of 128 bands, among which 8 are in thermal infrared region. The spectral resolution in visible region is about 10nm and the space resolution is about 3.5 meters. There is an artificial small target in the middle and top of image which occupies about 8 pixels (circled in Figure 7a). The CEM detection result for the target is shown in Figure 7b.

**Figure 8:**
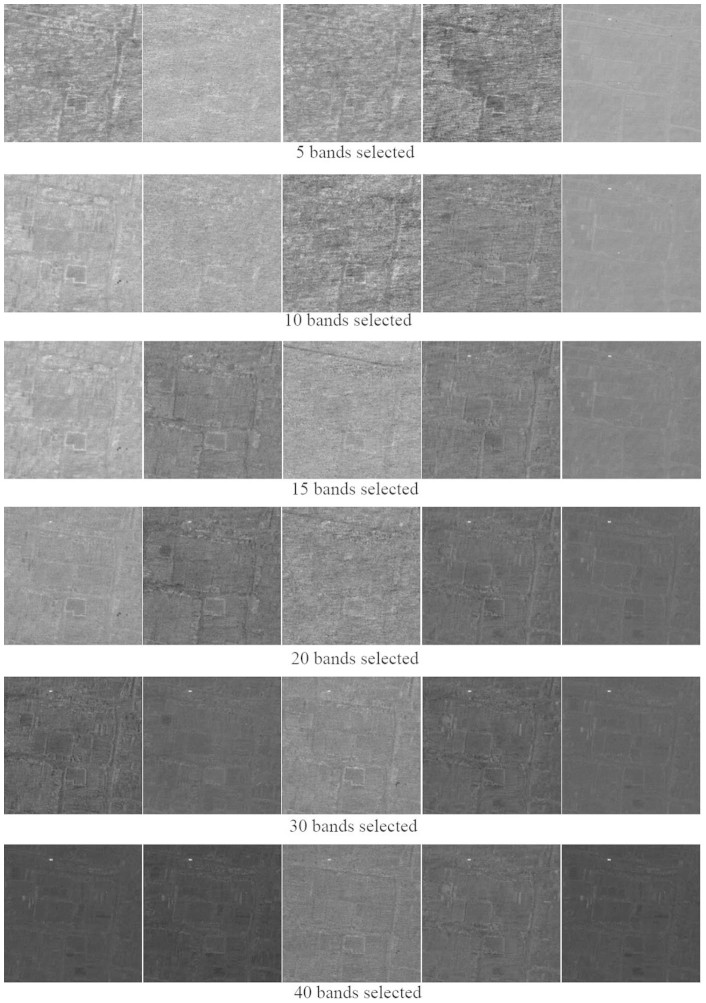
CEM results from bands selected by (from left to right) MEV, VNVBS, IDBS, ICABS and JSBS with different number.

We first selected 5, 10, 15, 20, 30 and 40 bands from the original 128 bands by these 5 methods respectively. Then the selected bands are used for CEM target detection, the corresponding results are shown in Figure 8.

From Figure 8 it can be seen that, only the bands selected by JSBS can be used to discriminant the target from background when the number of selected bands is small (<15). When the number of selected bands is large (for instance, 30 or 40), the band subsets selected by all the methods contains enough information to tell the targets from background. The CEM results are visually close to each other except for the deficient suppression to the background of IDBS selected bands. In addition, scanning from top to bottom, we can see that, all the methods show increasing detection performance if more bands are involved.

**Figure 9:**
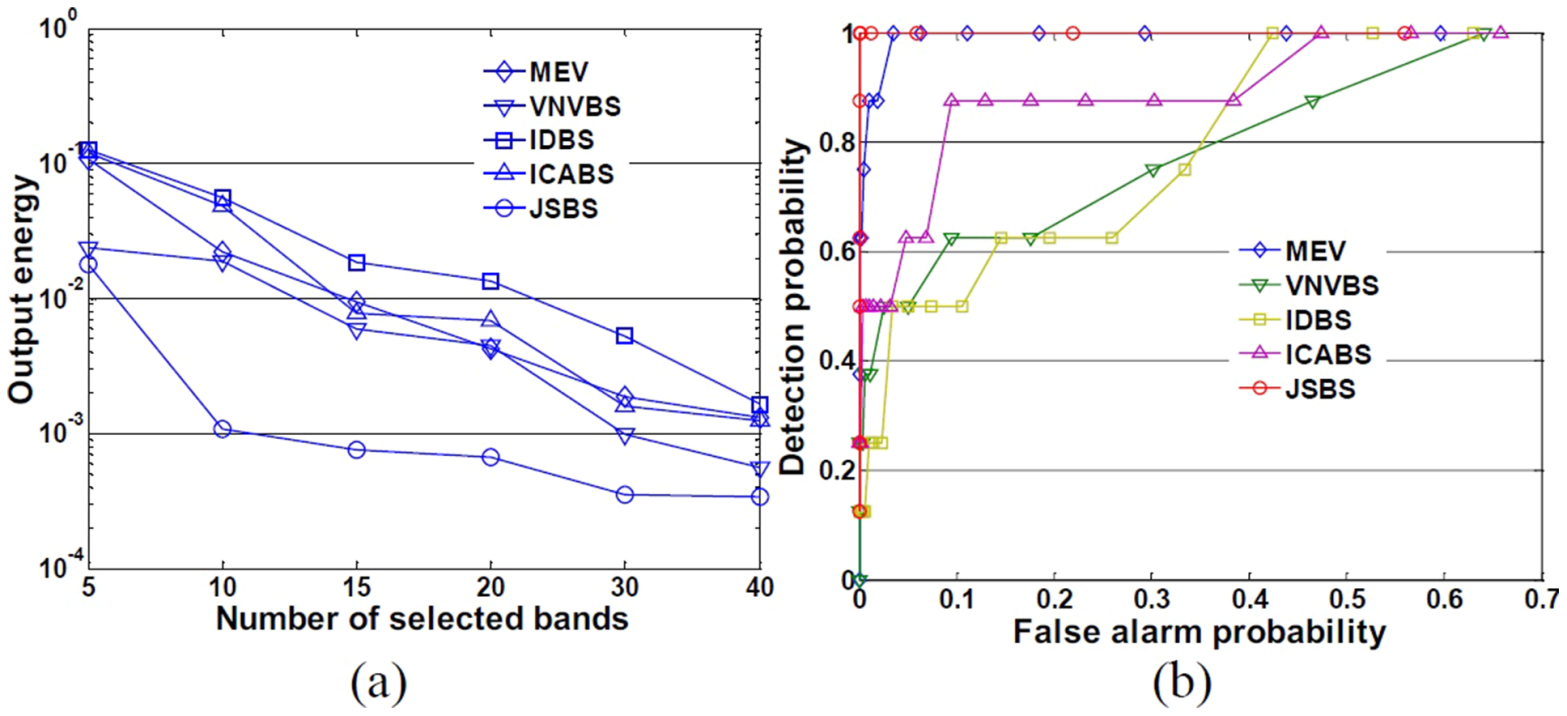
Quantitative measures: (a) output energy and (b) ROC curves (10 bands selected) for different methods.

In order to give further quantitatively evaluation, we use two indices, namely output energy and ROC. We can see that bands selected by JSBS always have the best performance in terms of artificial target detection since it always has the lowest output energy. JSBS shows much more superiority to the other methods, especially when the number of selected bands is small (for instance, 5 and 10). For instance, when 10 bands are selected, the ROC curves (Figure 9 b) indicate that JSBS outperforms the other methods in terms of target detection performance.

## Discussion

In this paper, we have proposed an unsupervised band selection method for small targets detection named joint skewness based band selection (JSBS). JSBS exploits the fact that, the non-Gaussian bands contains much information about small targets. In order to obtain the non- Gaussian as well as low correlated bands, we define joint skewness for band set based on the hyperdeterminant of third-order cumulant tensor. Then the hyperdeterminant has been approximately transformed into the matrix determinant by introducing the idea of HOSVD. The evaluation experiments demonstrate that, bands selected by JSBS are very effective in terms of artificial small target detection and always superior to those selected by the other BS methods.

It is noteworthy that, the singular values derived from HOSVD can only approximately correspond to the skewness. Therefore, the product of the singular values is the approximation of the joint skewness (hyperdeterminant of tensor). It needs to be further studied how to compute the joint skewness efficiently and accurately.

## Method

The presented JSBS is based on the third-order statistics of data and stimulated from the idea of MEV. Therefore, we briefly introduce the MEV method and basic concept of tensor at first.

### Covariance matrix determinant

**Figure 10:**
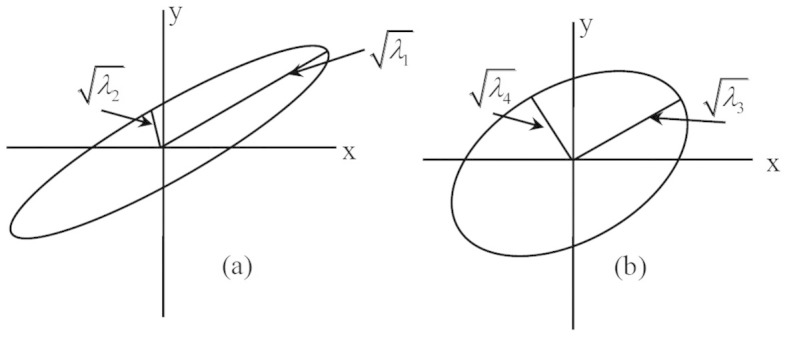
Different measure criterion for information, (a) the sum of square root of variances (information), with *λ*_1_ + *λ*_2_> *λ*_3_ + *λ*_4_ (b) the product of square root of variances, with *λ*_1_ × *λ*_2_ <*λ*_3_ × *λ*_4_.

Charles^17^ proposed an unsupervised band selection method (MEV) based on the second-order statistics of data. The criterion function used in MEV is the product of the variances (see Figure 10) in different principal directions. There MEV can be understood to select the subset with maximum joint second-order statistics (the product of variances in orthogonal directions). From geometric perspective, the product of the variances equals to the volume of the ellipsoid spanned in image space. On the other hand, from mathematical point of view, the criterion function can be transformed into the determinant of covariance matrix of bands. It has been demonstrated that this criterion discourages the selection of correlation band pairs.

### Tensor

**Figure 11:**
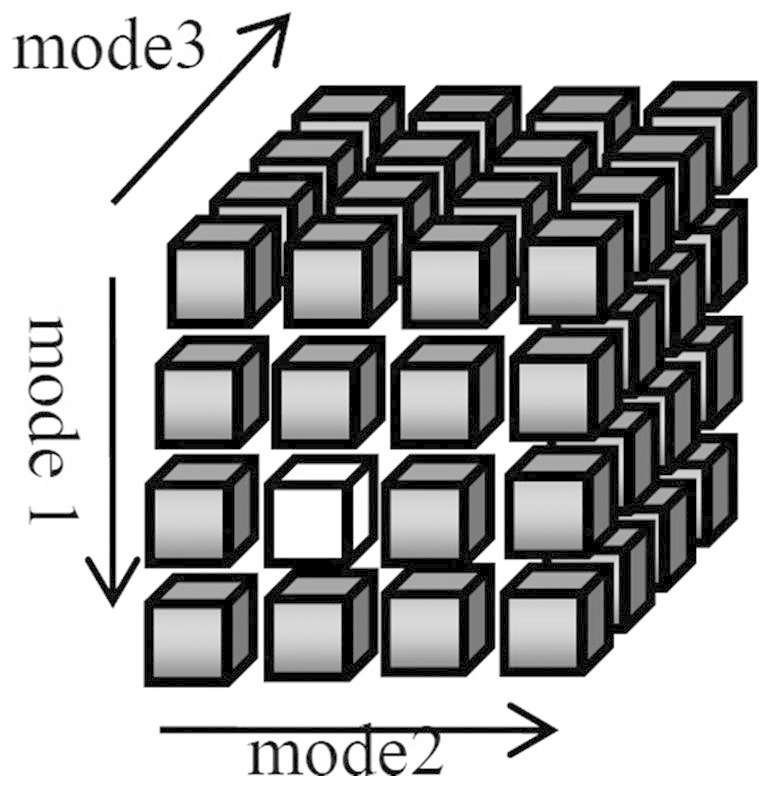
A sketch map of third-order tensor.

In this section, we give a brief introduction of tensor. A tensor is a multi-way array or multi-dimensional matrix. The order of a tensor is the number of dimensions, which is also known as ways or modes. The formal definition of tensor is as following: let *I*_1_, *I*_2_,…,*I_N_*,∈ N, a tensor 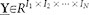 of order *N* is an *N*-way array where elements 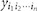 are indexed by *i_n_* ∈ {1,2,…,*I_n_*} for 1 ≤ *n* ≤ *N*^35^. A tensor is called super-symmetric if its entries are invariant with any permutation of their indices. Tensors are obviously generalizations of vectors and matrixes, for instance, as shown in Figure 11, a third-order tensor (or three-way array) has three modes (or indices or dimensions). The white element is denoted as *a*_321_.

**Figure 12:**
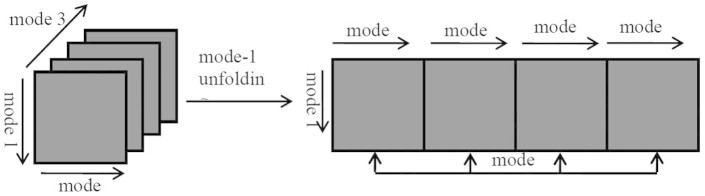
Sketch map of tensor unfolding.

Unfolding is an important operation for tensors which is also known as matricization or flattening. It is a process that reorders the elements of *N*-th order tensor into a matrix (see Figure 12). In general, there are *N* ways to reorder the elements of a *N*-th order tensor, called mode-i (*i* = 1,2,…,*n*) unfolding. But for third-order super-symmetric tensors 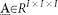, all the mode-i unfolding are the same, resulting in a same *I* × *I*^2^ matrix **A**_(*i*)_. More specifically, the mode-1 unfolding **A**_(1)_ of 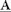 can be obtained as following:
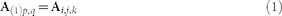
where *p* = *i*, *q* = (*k*−1)*i + *j*.

The determinant of a tensor is called hyperdeterminant^28^. The hyperdeterminant of a third-order tensor 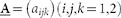 with size of 2 × 2 × 2 is given by^28^
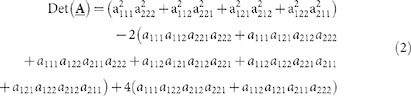
However, the hyperdeterminant of larger size third-order tensor is very hard to compute. In this paper, we introduce the idea of HOSVD for the calculation of hyperdeterminant.

Assume the hyperspectral image we obtained is of L-band and M pixels and is denoted as **X** = [**r**_1_,**r**_2_,…, **r***_M_*]*^T^*, where **r***_i_* = [*r_i_*_1_,*r_i_*_2_,…,*r_iL_*]*^T^* is the i-th pixel vector. For the sake of convenience, we first remove the mean value of each band in **X**. For band selection, we need to select a group of bands with maximum overall skewness. Therefore, we have to investigate the joint skewness for the band set rather than the skewness for single band. According to the authors' knowledge, there is no definition for JS. In the following section, we give a definition for JS based on third-order tensors.

### Joint skewness for multivariate data

Charles^17^ uses the determinant of covariance matrix of the data to characterize the joint second-order statistics (variance) and claims that this determinant discourages the selection of highly correlated band pairs. Meanwhile, it is further proved that this determinant corresponds to the joint entropy of the bands under the assumption of Gaussian distribution.

**Figure 13:**
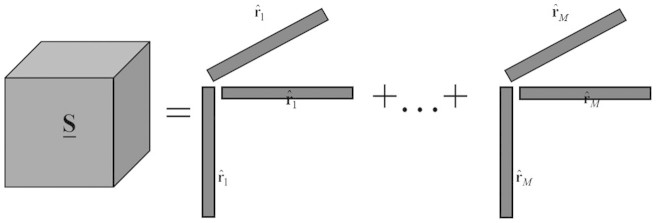
sketch map of the calculation of S (coefficient 1/*M* is ignored).

Coskewness tensor is the natural generalization of covariance matrix to third-order statistics^36^,^37^,^38^. In order to calculate the coskewness tensor, the image needs to be centralized and whitened firstly by
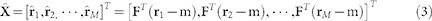
where 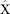 is the whitened data set, **F** = **ED**^−1/2^, is the whitening matrix (**E** and **D** are the eigenvector matrix and the corresponding eigenvalue matrix for covariance matrix of **X**) and **m** is the mean vector of the image. The corresponding coskewness tensor of an image, denoted as **S**, is defined as
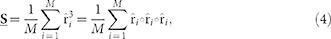
where the 3-way outer product 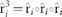 is a rank-1 third-order tensor with L dimensions. In fact, each element of 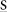 can be calculated by
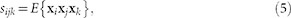
where **x***_i_* is the vector reshaped by the i-th band and 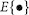 is the expectation operator. Figure 13 shows a sketch map of the calculation of 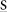.

Since the determinant of covariance matrix corresponds to the overall second-order statistics, would the hyperdeterminant of coskewness tensor characterize the overall third-order statistics? If this speculation holds, it may implicate great applicable value for hyperspectral band selection. Based on this assumption, we define the joint skewness for multivariate data as
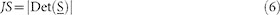
where 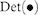 is the hyperdeterminant operator and 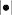 is absolute operator.

However, since data whitening is needed in advance to compute coskewness tensor, 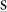 needs to be frequently calculated for different band combinations if we use equation (6) as BS criterion. Therefore, we decide to adopt another expression for JS which does not require data whitening. The skewness of an arbitrary random vector **v**, *skew*(**v**) is defined as the third-order standard moment, which is also the ratio of third-order cumulant to 1.5 power of the second-order cumulant as following:
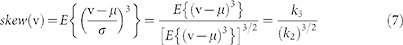
where *σ* and *μ* are the standard deviation and mean value of **v**, and *k_i_* is the i-th-order cumulant of **v**.

Combining equation (6) and (7), we redefine the joint skewness for multivariate data as
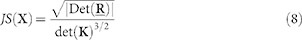
where 
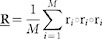
 is the third-order cumulant tensor and 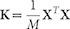 is the covariance matrix for hyperspectral image **X**. Compared to the repetitious calculation of sub-coskewness tensor **S** in band selection, 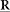 only needs to be computed once since it does not involve whitening of data.

Based on (8), we expect to obtain the bands with not only large overall skewness but also low correlation. Unfortunately, the computation of hyperdeterminant of third-order tensor (i.e. 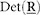) with large size (large than 2×2×2) is quite difficult^28^.

Interestingly, we can transform the hyperdeterminant of tensor into the determinant of matrix by introducing the idea of HOSVD in the following, which makes it much easier to compute. This is the key point of our method.

Geng et al.^36^ proposed a novel artificial small targets detection method by performing HOSVD to coskewness tensor. Interestingly, it is found that the singular values derived from HOSVD highly correspond to the skewness in singular vector directions. This conclusion plays a quite crucial role for JSBS.

We speculate that the hyperdeterminant of coskewness tensor should correspond to the product of the skewness in all the singular directions since we know the determinant of covariance matrix equals to the product of the square root of the variance of all the principal directions. Combing this speculation with the conclusion about the relationship between singular values and skewness drawn in^36^, we consider that, could we approximate the hyperdeterminant of tensor by the product of the singular vales derived from HOSVD? If this assumption holds, then the calculation of hyperdeterminant of third-order tensor can be transformed into the computation of determinant of matrices. Fortunately, after conducting related experiments on simulated data (section 4.1), we find that the product of the singular values derived from HOSVD has a strong positive relation (correlation coefficient is about 0.97) with the hyperdeterminant of coskewness tensor. As a result, we have transformed the hyperdeterminant problem of tensor to the determinant problem of matrix by exploiting the property of HOSVD.

Specifically, the HOSVD to the third-order tenor 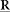 can be performed as follows: first, we unfold tensor 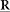 into matrix **R**_(*n*)_ in mode-n way (the size of **R**_(*n*)_ is *L* × *L*^2^). Then, perform SVD of matrix to **R**_(*n*)_. The n mode-n unfoldings of 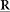 are totally the same since it is a super-symmetric tensor, i.e. **R**_(1)_ = **R**_(2)_ = … = **R**_(*L*)_. It can be easily verified that the singular values (*λ*_1_, *λ*_2_,…,*λ_L_*) derived from SVD to **R**_(*n*)_ satisfy
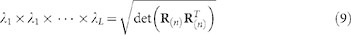
Consequently, equation (8) can be approximately transformed into the following matrix operation,
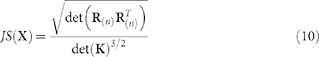
By now we have defined the joint skewness for multivariate data as the hyperdeterminant of coskewness tensor and approximately transformed it into simple matrix operations as shown in equation (10). The evaluation experiments based on real hyperspectral data (section 4.2 and 4.3) verify the equation (10) works well. The following problem is how to find the band subset that has maximum value of (10) which is known as band subset selection.

### Search Strategy

It is a NP hard problem for band subset search. Although Narendra et al.^39^ have proposed an efficient subset search method, branch and bound (BB), it is only suitable for monotonous criterion functions. As to the common criterion functions, the search of optimal subset is computationally prohibitive when the number of bands is over a few tens (which obviously includes hyperspectral data)^40^. Therefore, some methods that are fast to implement and can provide acceptable band subset (not necessarily the best one) are required. Many subset search methods that trade off efficiency against accuracy have been reported in literatures, for instance,^41^,^42^,^43^,^44^. Among these methods, SBS^41^ is one of the most widely used ones due to its simplicity and effectiveness.

SBS is “top-down” method which starts with full band set and followed by removing the redundant bands one-by-one. In each iteration, the subset (constructed by removing one band from original band set) with maximum (or minimum, depending on the selection criterion) value of criterion function is reserved. It is continued until the number the left bands meets the requirement. We employ SBS as the subset search method for JSBS. The pseudo code in Matlab style is listed in the following:

### Algorithm: JSBS

Input: Observations X = [**X**_1_,**X**_2_,…,**X***_L_*], where **X***_i_* = [*x*_1*i*_,*x*_2*i*_,…,*x_Mi_*]*^T^* is the column vector reshaped by the i-th band and the number of selected bands p.

1. Initialization
  - remove the mean value of each band;
  - Compute the second-order and third-order cumulant matrix (tensor) which are denoted as **K** and 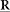 respectively.
  - set the selected band index set Φ = {1,2,...,*L*} and indicating variable k = L.
  %% Main loop While (k > p)
2. Remove each band in Φ tentatively and calculate the joint skewness (denoted as JS), for the corresponding left band. More specifically, For i = 1 to k
  - Construct K^(i)^ by removing the i-th row and the i-th column of K, construct 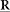
^(i)^ by removing the i-th horizontal slice, i-th lateral slice and the i-th frontal slice of 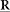.
  - Unfold 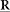
^(i)^ into matrix 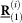 (since **R**_(1)_ = **R**_(2)_ = ... = **R**_(*L*)_).
  - Calculate the joint skewness for corresponding left bands by (7),
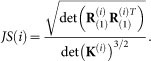
 Endfor
3. Determine the band to be removed, 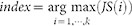.
4. Remove the corresponding index from selected band index set, the new index set is Φ = {1,2, …,index−1,index + 1,…,L}.
5. Update K and 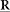, respectively by K = K(index), 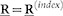.
6. k←k−1 Endwhile
7. Output: Φ = {a1,a2,…,a*_p_*} are the final selection band indices.

## Author Contributions

X.G. conceived the idea. X.G., K.S., L.J. and Y.Z. designed and performed the experiments and analyzed the data. X.G., K.S., L.J. and H.T. wrote the main manuscript text. All authors reviewed the manuscript.

## Figures and Tables

**Table 1:** The specifications of the simulated data

	Band 1	Band 2	Band 3	Band 4
Variance	9.1649	9.0257	1.0473	0.6983
Skewness	0.0993	0.0832	0.6078	1.0887
Correlation coefficient	0.0262		0.9914	

**Table 2:** Band selection results from different methods

Method	MEV	IDBS	ICABS	JSBS
Selected bands	1,2	3,4	3,4	2,4
